# Phylodynamics of Yellow Fever Virus in the Americas: new insights into the origin of the 2017 Brazilian outbreak

**DOI:** 10.1038/s41598-017-07873-7

**Published:** 2017-08-07

**Authors:** Daiana Mir, Edson Delatorre, Myrna Bonaldo, Ricardo Lourenço-de-Oliveira, Ana Carolina Vicente, Gonzalo Bello

**Affiliations:** 1 Laboratório de AIDS e Imunologia Molecular, Instituto Oswaldo Cruz, Fiocruz, Rio de Janeiro Brazil; 2 Laboratório de Biologia Molecular de Flavivírus, Instituto Oswaldo Cruz, Fiocruz, Rio de Janeiro Brazil; 3 Laboratório de Mosquitos Transmissores de Hematozoários, Instituto Oswaldo Cruz, Fiocruz, Rio de Janeiro Brazil; 4 Laboratório de Genética Molecular de Microrganismos, Instituto Oswaldo Cruz, Fiocruz, Rio de Janeiro Brazil

## Abstract

Yellow fever virus (YFV) strains circulating in the Americas belong to two distinct genotypes (I and II) that have diversified into several concurrent enzootic lineages. Since 1999, YFV genotype I has spread outside endemic regions and its recent (2017) reemergence in non-endemic Southeastern Brazilian states fuels one of the largest epizootic of jungle Yellow Fever registered in the country. To better understand this phenomenon, we reconstructed the phylodynamics of YFV American genotypes using sequences from nine countries sampled along 60 years, including strains from Brazilian 2017 outbreak. Our analyses reveals that YFV genotypes I and II follow roughly similar evolutionary and demographic dynamics until the early 1990s, when a dramatic change in the diversification process of the genotype I occurred associated with the emergence and dissemination of a new lineage (here called modern). Trinidad and Tobago was the most likely source of the YFV modern-lineage that spread to Brazil and Venezuela around the late 1980s, where it replaced all lineages previously circulating. The modern-lineage caused all major YFV outbreaks detected in non-endemic South American regions since 2000, including the 2017 Brazilian outbreak, and its dissemination was coupled to the accumulation of several amino acid substitutions particularly within non-structural viral proteins.

## Introduction

Yellow fever virus (YFV) is the causative agent of yellow fever (YF), a severe acute disease of historical importance that remains a major public health problem in endemic regions of South America and Africa^1, 2^. YFV was probably introduced in the Americas from Africa around 300–400 years ago^3, 4^, coinciding with the slave trade period, and caused numerous YF urban epidemics in the continent until the early 20^th^ century^1, 2^. Since 1950, transmission of YFV in the Americas has been mostly maintained in a sylvatic cycle involving New World primates and mosquitoes of the genera *Haemogogus* and *Sabethes*. YFV is highly pathogenic for some New World primates and epizootics occur at rather regular intervals (~5–10 years) in a particular geographic region, sometime coinciding with sporadic outbreaks of YF in unvaccinated humans living in forested and surrounding rural areas^1, 2^.

Most epizootic/epidemic outbreaks of YFV reported in Brazil during the second half of the 20^th^ century were mainly restricted to the endemic Northern (Amazon) and Central-Western regions; but since 1999 the YFV spread outside the established endemic regions affecting an increasing number of humans and non-human primates in the Southeastern and Southern Brazilian regions^5–7^. The most recent outbreak of YF outside the endemic region began in December 2016 and affected non-human primates and unvaccinated human populations from rural areas of all four Southeastern Brazilian states (Minas Gerais, Espírito Santo, Rio de Janeiro and São Paulo), resulting in the largest epizootic/epidemic of jungle YF registered in Brazil over the last 50 years. Between December 2016 and April 2017, a total of 473 epizootics of non-human primates and 623 humans cases with 209 deaths (case-fatality, 33.5%) were confirmed, mostly concentrated in Southeastern Brazilian states^8^. This corresponds to 79% of the total number of human confirmed cases of YFV across all Brazilian regions between 1980 and 2015^9^.

YFV strains currently circulating in South America branch within two distinct genotypes (I and II) that probably arose around the second half of the 19^th^ century^10^. These genotypes have diversified into several concurrent enzootic lineages that appear to persist and evolve within distinct geographic areas of Brazil, Bolivia, Peru, Trinidad and Tobago and Venezuela for long time periods^10–15^. While *in situ* evolution appears to be the predominant mechanism of YFV maintenance in South America and the Caribbean, some studies detected occasional YFV migrations between different American countries on long-time scales^13, 15^ and others showed that YFV outbreaks were associated with the emergence of new lineages that replaced those causing previous outbreaks, as observed in the recent Brazilian epidemics (2000–2008)^10, 12^. These observations suggest that lineage re-introduction and replacement may have been important factors shaping the long-term evolution and emergence of new YFV outbreaks in the Americas; but their relevance could have been underestimated because the paucity of viral sequences representative of all countries sampled over long-time scales.

The objective of this study was to gain new insights about the impact of *in situ* evolution and lineage replacement as driving forces of the recent expansion of YFV to non-endemic South American regions. To do this, we reconstruct the long-term evolutionary, demographic and phylogeographic dynamics of dissemination of major YFV lineages circulating in the Americas using a much more comprehensive and up-to-date dataset than in previous studies. Our dataset comprised 137 YFV sequences sampled from nine South American and Caribbean countries between 1954 and 2017, including the two recently described YFV sequences from the Brazilian 2017 outbreak^16^. Additionally, we reconstructed the non-synonymous substitutions fixed along the entire genome of the ancestral virus from which the new YFV outbreaks occurring in the Americas evolved.

## Results

### Spatial and temporal diversification of YFV genotypes I and II in the Americas

The Maximum Likelihood (ML) phylogenetic analysis of YFV prM/E gene sequences revealed that all American isolates (not related to vaccine strains) segregate in two reciprocally monophyletic clusters corresponding to genotypes I (*n* = 100) and II (*n* = 37) (Supplementary Fig. S1). Genotype I comprises most YFV sequences from Brazil (95%), Colombia (100%), Venezuela (100%), and Trinidad and Tobago (92%); whereas genotype II comprises all Peruvian sequences included in our analysis. In order to gain insight into the temporal and spatial dissemination of YFV in the Americas, prM/E sequences from each genotype were analyzed using a Bayesian MCMC phylogeographic approach. The mean evolutionary rates here estimated for YFV genotypes I and II were very similar among each other (~5 × 10^−4^ subs./site/yr) and fully consistent with those previously reported for YFV prM/E sequences of American and/or African origin (Table 1)^3, 13, 17, 18^.

**Table 1:** Bayesian estimates of evolutionary parameters of the YFV American genotypes.

**Region**	**Genotype**	µ* (substitutions site^−1^ year^−1^)	Coeficient of variation*	**T**_MRCA_*
prM/E	I	5.2 × 10^−4^ (3.4 × 10^−4^–7.4 × 10^−4^)	0.74 (0.41–1.11)	1908 (1870–1936)
	II	4.4 × 10^−4^ (2.0 × 10^−4^–7.5 × 10^−4^)	0.76 (0.37–1.19)	1920 (1867–1958)
CDS**	I	3.4 × 10^−4^ (2.5 × 10^−4^–4.5 × 10^−4^)	0.14 (1.17 × 10^−4^–0.28)	1933 (1916–1950)
	II			1907 (1874–1939)

*The 95% HPD is displayed in parentheses. **Complete coding sequence.

**Figure 1:**
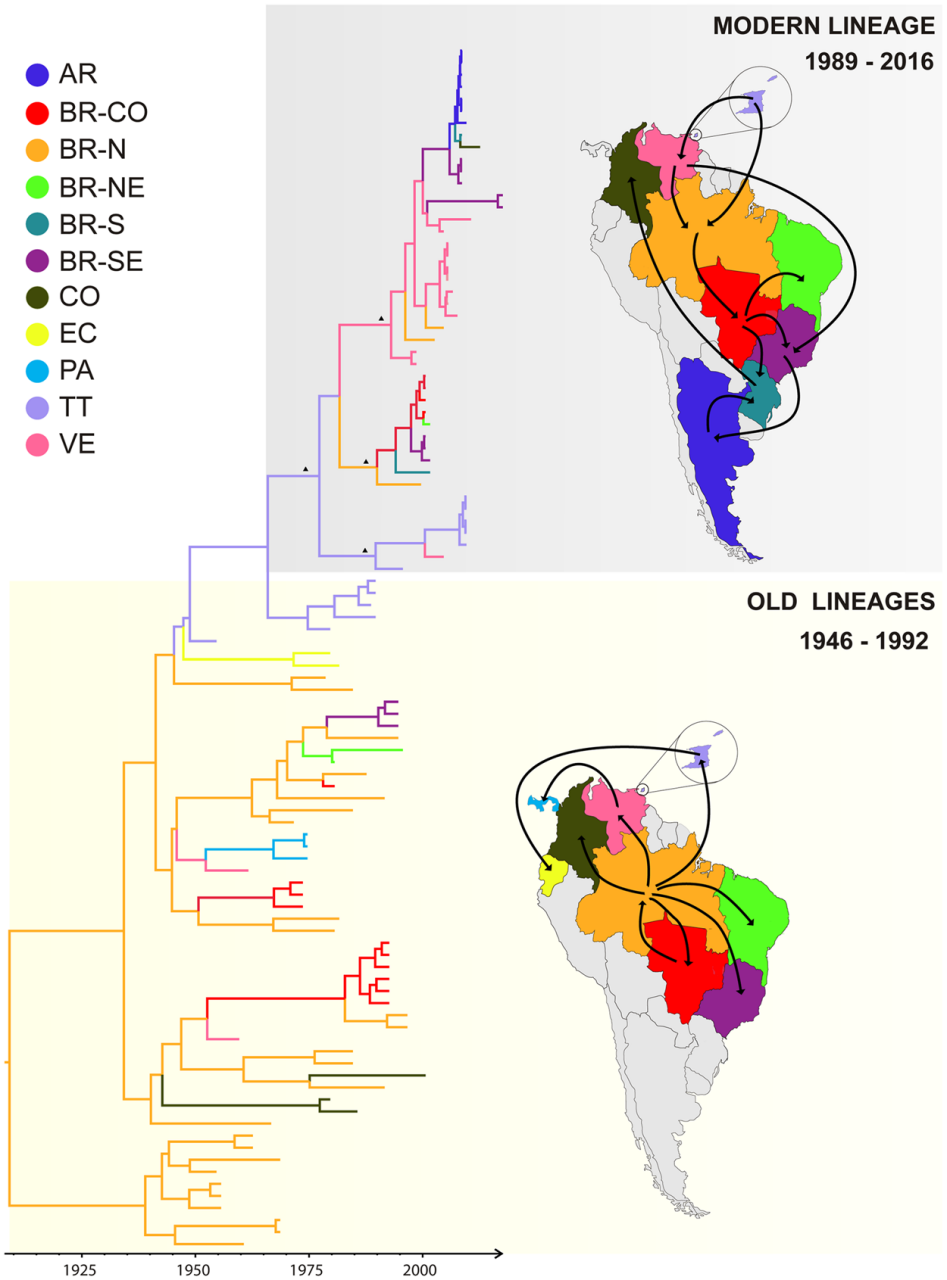
Time-scaled Bayesian phylogeographic MCC tree of the YFV genotype I prM/E gene sequences. Branches are colored according to the most probable location state of their descendent nodes as indicated at the legend (top left). Key ancestral nodes of modern-lineage subclades with high posterior probability support (clade credibility >0.99) are indicated with dark full triangles. All horizontal branch lengths are drawn to a scale of years. The tree is automatically rooted under the assumption of a relaxed molecular clock. (AR: Argentina, BR-CO: Brazil Central-West, BR-N: Brazil North, BR-NE: Brazil Northeast, BR-S: Brazil South, BR-SE: Brazil Southeast, CO: Colombia, EC: Ecuador, PA: Panama, TT: Trinidad and Tobago, VE: Venezuela). Viral migration events occurred within the old lineages (1946–1992) and the modern one (1989–2016) are summarized in the maps. Lines between locations represent branches in the Bayesian MCC tree along which location’s transitions occur. Maps were created with CorelDraw from templates obtained from d-maps.com. (South America: http://www.d-maps.com/carte.php?num_car=28522&lang=en; and Brazil: http://www.d-maps.com/carte.php?num_car=16016&lang=en).

The spatiotemporal reconstruction of YFV genotype I suggests that this American lineage likely originated in the Northern Brazilian region (posterior state probability (*PSP*) = 0.95) at around 1908 (95% HPD: 1870–1936) (Fig. 1). Between 1940 and 1990, YFV genotype I spread from Northern Brazil (*PSP* > 0.75) to other Brazilian regions (Central-Western, Northeastern and Southeastern) as well as to other American countries (Colombia, Venezuela and Trinidad and Tobago) at multiple times. The analysis suggest that during these decades there were also secondary viral disseminations from Venezuela (*PSP* = 0.45) to Panama, from Trinidad and Tobago (*PSP* = 0.69) to Ecuador, and from Brazilian Central-Western (*PSP* > 0.66) to Brazilian Northern region, although the supporting *PSPs* values of those migrations were relatively low. Until the middle 1990s, several YFV genotype I lineages (designated as old-lineages) co-circulated and diversified while spread through different American countries and Brazilian regions.

At the middle 1990s, a dramatic change in the genetic diversity of the YFV genotype I occurred with the emergence of a new lineage (designated as modern-lineage) that replaced old-lineages circulating in the previous decades. The modern-lineage comprises all YFV genotype I sequences isolated in the Americas after 1996, with the exception of one sequence isolated in Colombia in 2000 that branched among old-lineages. Our phylogeographic analysis supports that the modern-lineage probably arose in Trinidad and Tobago (*PSP* = 0.72) at 1977 (95% HPD: 1964–1987), where it continued circulating until the most recent survey performed in 2009. The modern-lineage did not remain contained to Trinidad and Tobago, but was concurrently disseminated to Northern Brazil (*PSP* = 0.52) and to Venezuela (*PSP* = 0.83) at 1989 (95% HPD: 1981–1996) and 1992 (95% HPD: 1986–1997), respectively. From Northern Brazil, a modern-sublineage rapidly spread southward reaching the Central-Western region in 1993 (95% HPD: 1987–1998) and non-endemic Brazilian regions (Northeastern, Southeastern and Southern) at later times. This modern-sublineage was associated to the 2000–2001 Brazilian outbreaks, but there is no evidence of further circulation of this subclade after that time in the country. The modern-lineage strain introduced in Venezuela continued to circulate and evolve in this country until 2010 (year of the most recent sample available), generating another sublineage that was independently disseminated from Venezuela (*PSP* > 0.90) into Northern Brazil (originating sporadic human cases) and into Southeastern Brazil. Its first introduction into the Southeastern Brazilian region was estimated at 2005 (95% HPD: 2002–2007), driving the 2008–2009 outbreak that later spread to Southern Brazil and Northern Argentina. An independent dissemination of the modern-lineage from Venezuela into Southeastern Brazil seems to have originated the recent 2017 Brazilian outbreak. The most recent common ancestor (MRCA) of the two Brazilian YFV strains from 2017 was traced to 2016 (95% HPD: 2012–2017).

**Figure 2:**
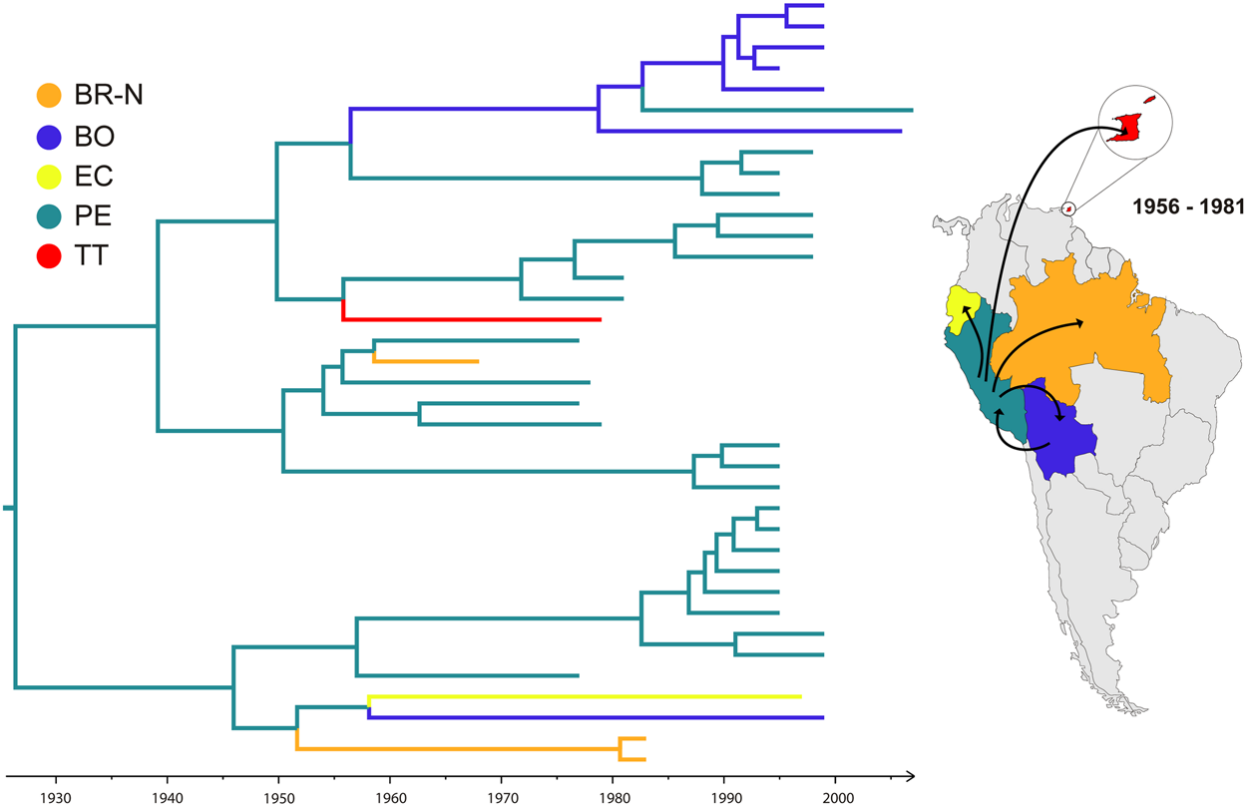
Time-scaled Bayesian MCC phylogeographic tree of the YFV genotype II prM/E gene sequences. Branches are colored according to the most probable location state of their descendent nodes as indicated at the legend (top left). All horizontal branch lengths are drawn to a scale of years. The tree is automatically rooted under the assumption of a relaxed molecular clock. (BR-N: Brazil North, BO: Bolivia, EC: Ecuador, PE: Peru, TT: Trinidad and Tobago). Viral migration events occurred within genotype II (1956–1981) are summarized in the maps. Lines between locations represent branches in the Bayesian MCC tree along which location’s transitions occur. Maps were created with CorelDraw from templates obtained from d-maps.com. (South America: http://www.d-maps.com/carte.php?num_car=28522&lang=en; and Brazil: http://www.d-maps.com/carte.php?num_car=16016&lang=en).

Spatiotemporal reconstruction of YFV American genotype II dissemination, suggests that this lineage likely originated in Peru (*PSP* = 0.96) at 1920 (95% HPD: 1867–1958) (Fig. 2). In the following years, YFV genotype II diversified into several lineages that persist in Peru for several decades, without evidence of lineage replacements over time. YFV genotype II was sporadically disseminated from Peru (*PSP* > 0.70) to other locations (Bolivia, Ecuador, Northern Brazil and Trinidad and Tobago) at multiple times. Most of those introductions seem to have resulted in dead end infections, with the exception of a genotype II variant probably introduced into Bolivia at 1973 (95% HPD: 1942–1992) that was locally spread and remained circulating in this country until 2006. We detected only one reintroduction of genotype II into Peru, probably from Bolivia (*PSP* > 0.59).

### Population dynamics of YFV genotypes I and II in the Americas

**Figure 3:**
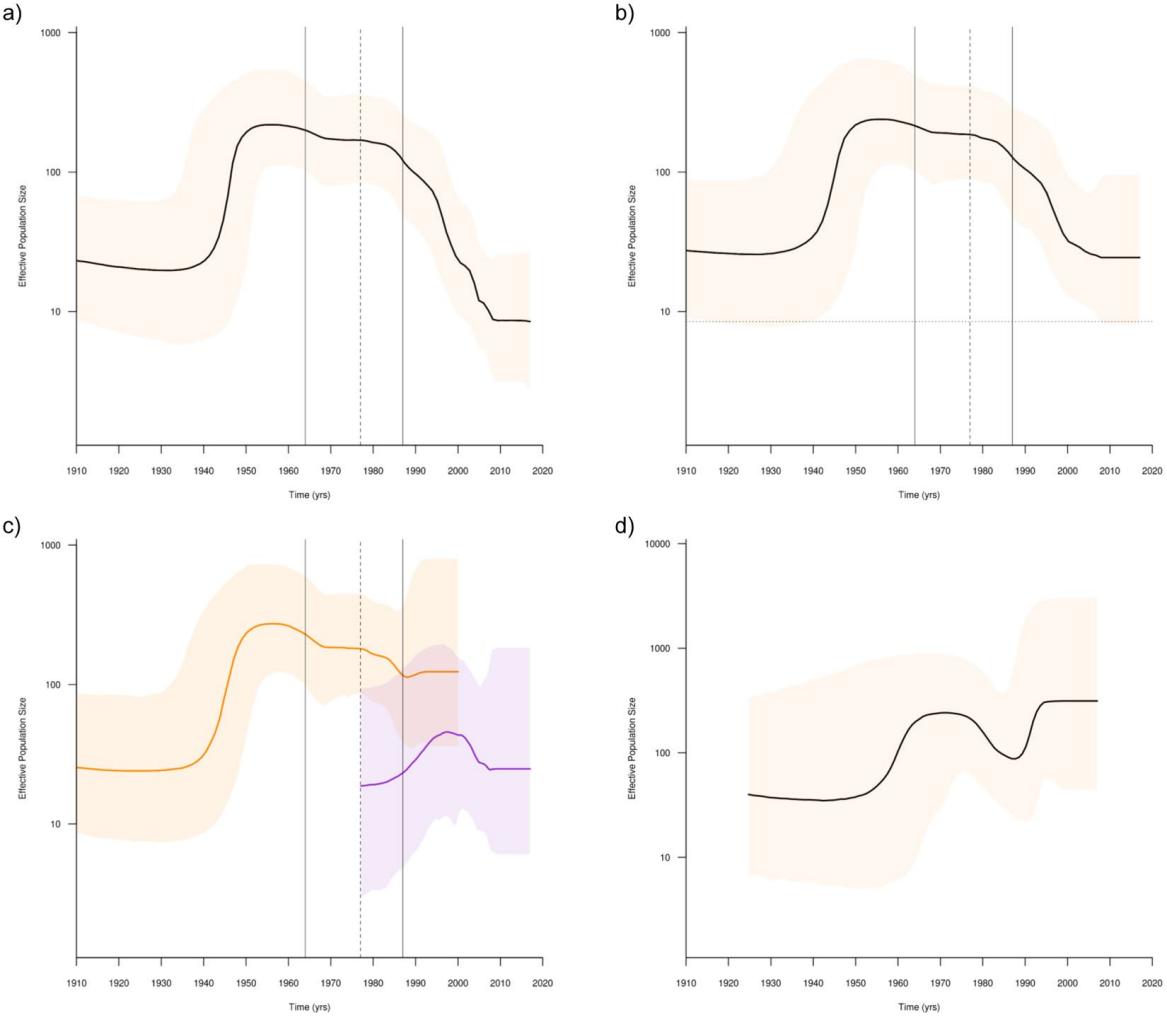
Demographic history of YFV genotypes I and II in the Americas. Mean estimates of the effective number of infections (*N*_e_) (solid line) are shown together with the 95% HPD intervals (shaded area) of the Bayesian skyline for whole Genotype I dataset (**a**), Genotype I subsampled dataset (**b**), Genotype I old-lineages (orange line) and modern-lineage (purple line) (**c**), and Genotype II (**d**). Vertical segmented and continuous lines in demographic plots of panels a, b and c represents the estimated mean T_MRCA_ for the genotype I modern-linage and its 95% HPD interval, respectively. Horizontal segmented line in panel b represents the median *Ne* value at present estimated with the complete Genotype I dataset. The vertical axes represent the estimated *N*_e_ on a logarithmic scale. Time scales are in calendar years.

The demographic history reconstructions revealed differences in the population growth dynamics of both YFV American genotypes. The Bayesian skyline plot (BSP) analysis suggest that YFV genotype I displayed a phase of exponential growth between 1935 and 1950, followed by a stabilization of the effective population size (*N*_e_) between 1950 and 1985, and a subsequent drastic reduction between 1985 and 2010 that roughly coincides with the emergence and dissemination of the modern-lineage through South America (Fig. 3a). To assess whether the observed genotype I demographic pattern has been influenced by the confounding effect of geographic population structure due to higher sampling density at recent YFV outbreaks, we removed epidemiologically related modern-lineage sequences sampled at the same YFV outbreaks leaving only one representative per location and year (Supplementary Fig. S2). The demographic analysis of this YFV genotype I subset also showed a reduction in the *N*_e_ after 1985, but of lower magnitude than that observed for the complete genotype I dataset (Fig. 3b). Next, we tested the potential impact of different epidemic dynamics among YFV lineages on the inferred demographic pattern of genotype I by performing separate BSP analyses for the old and modern (with sub-sampling) lineages. Although the demographic reconstruction indicates some overlap between the 95% HPD intervals for the estimated mean *N*_e_ for the modern-lineage and the paraphyletic old-lineages, the overall trend suggests that the modern-lineage has so far exhibited a lower *N*_e_ than the paraphyletic old-lineages between 1980 and 2000 (Fig. 3c). These new BSP analyses also uncover that the estimated mean *N*_e_ for the modern-lineage displayed a steady increase during the 1980s and 1990s, although this observation should be interpreted with caution because the very large 95% HPD intervals. The BSP analysis of YFV genotype II indicate that this lineage displayed a short exponential growth phase between 1955 and 1965, after which the *N*_e_ remains roughly stable up to the most recent time (Fig. 3d). Only a small fluctuation (reduction and subsequent increase) in the *N*_e_ of the genotype II was observed between 1980 and 1995, coinciding with the introduction and dissemination of this lineage in Bolivia. Although this results support a trend towards increasing *N*_e_ for YFV genotype II over time, a constant size model is also contained within the very large boundaries of the 95% HPD interval. To discriminate between these alternative hypotheses, demographic models allowing fluctuations in *N*_e_ over time were compared with the constant size model. The model comparison revealed that both Bayesian skyline and exponential growth models outperformed the constant size model (Supplementary Table S1).

### Genetic signatures at ancestral nodes on the YFV genotype I phylogeny

**Figure 4:**
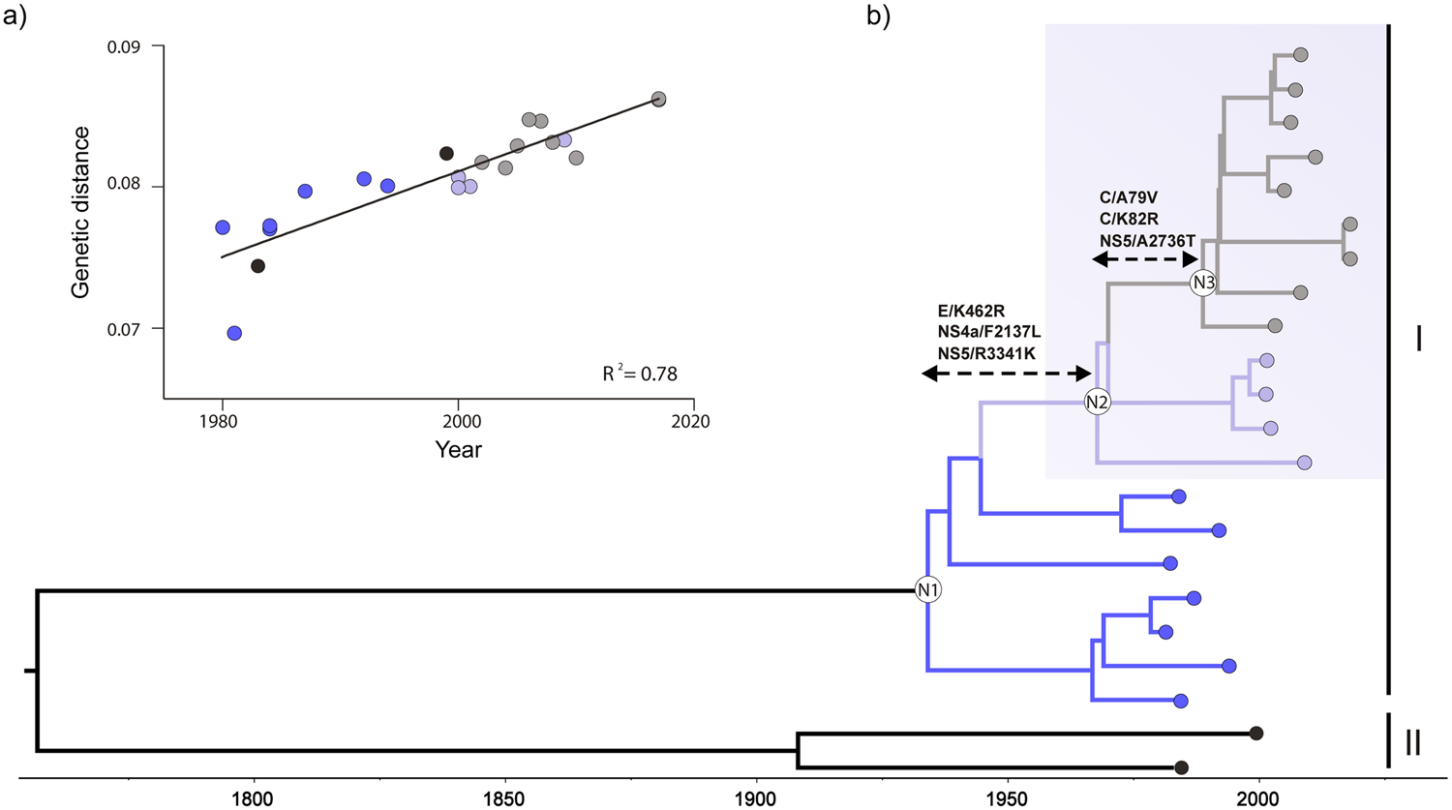
Evolutionary analysis of YFV genotype I and II complete coding sequences (CDS). (**a**) Correlation between the sampling date of each sequence and the genetic distance of that sequence from the root of a maximum likelihood phylogeny of the YFV CDS (R^2^ = 0.78) is displayed (top left). (**b**) Time-scaled Bayesian MCC tree of YFV CDS. The shaded box highlights the modern-lineage clade. Genotype I branches corresponding to old, modern-basal and modern-subclade 1E lineages were colored in blue, light purple and gray, respectively. Reconstructed ancestral key nodes of all genotype I sequences (N1), all modern-lineage sequences (N2) and the modern subclade 1E (N3) are indicated. Inferred amino acid substitutions between ancestral key nodes are shown in relation to the polyprotein positions.

Despite the low number of YFV complete genomes available for genotypes I and II, there was observed a very good correlation between sequence sampling year and divergence from the root, thus supporting the molecular clock signal of this dataset (Fig. 4a). The overall time-scale estimated from YFV complete coding sequences (CDS) and prM/E datasets were roughly comparable (Table 1). Ancestral CDS were reconstructed at the key nodes of YFV genotype I phylogeny corresponding to the MRCA of: all genotype I sequences (N1), all modern-lineage sequences (N2), and the modern-sublineage comprising the most recent sequences from Brazil and Venezuela, previously designated as subclade 1E (N3)^10, 12^ (Fig. 4b). These analyses showed the fixation of three non-synonymous mutations over the 35 years interval between nodes N1 (1934 [95% HPD: 1916–1950]) and N2 (1968 [95% HPD: 1957–1979]), while three other non-synonymous mutations were detected over the much shorter time interval between nodes N2 and N3 (1989 [95% HPD: 1983–1994]) (Fig. 4b and Supplementary Table S2). This analysis also revealed that several amino acid mutations arose during the recent evolution of the modern subclade 1E in Brazil and Venezuela, mainly (91%) within non-structural proteins (Supplementary Table S3). It is noteworthy that the most recent sequences from 2017 Brazilian outbreak displayed the highest number of amino acid differences (n = 9) with respect to ancestor N3, most of which were described as unique genetic signatures of those sequences^16^.

## Discussion

YFV surveillance remains a critical public health issue in South America and the Caribbean, as YF outbreaks are continuously occurring in those regions. The YFV outbreaks occurring in Brazil after 2000 seem to be following a path towards the Southern/Southeastern regions^5^, migrating from endemic areas to areas in which there were no YF cases for decades and where YFV vaccine is not routinely administered, and hence, where most human population is susceptible to the virus. The current reemergence of YFV in the Southeastern Brazilian states resulted in the largest and most devastating epizootic/epidemic of jungle YF registered in Brazil over the last 50 years, an unexpected scenario that had also been observed in the YF epizootic-epidemic waves in Brazil between the 1930–1940 decades^19^. As ecologic monitoring of YFV is extremely difficult, an alternative is to use viral phylodynamics to gain insight into the spatiotemporal dispersal pattern of this virus. Assessing the dynamics of YFV spread and maintenance in the Americas comprises a paramount tool to control and prevent future outbreaks.

Previous studies supported that YFV American genotypes have diversified into several concurrent enzootic lineages that appear to persist and evolve within distinct geographic areas for long time periods^10–15^. Thus, *in situ* evolution was pointed as the key mechanism shaping the epidemic dynamics of YFV in America, rather than the long-distance spread through epizootic waves of infection. In accordance with this hypothesis, we detected the co-circulation of several YFV genotype II lineages that have remained mostly restricted to Peru, with no evidence of lineage replacements over time. Long-distance YFV genotype II migrations were sporadic and most end with apparent lineage extinction, with exception of one introduction in Bolivia that was followed by local maintenance of the virus for a long period (>30 years). The causes of the restricted spread of YFV genotype II outside Peru remain unclear, but cross-immunity between antigenic similar genotypes may certainly function as a barrier for dissemination of YFV genotype II into geographic areas where YFV genotype I already circulates. The Andean mountains may also pose a physical barrier for the spread of YFV genotype II outside of Peru.

Lineage replacement and long-distance migrations between countries, by contrast, played a crucial role in the long-term evolution and widespread disseminations of YFV genotype I. Several enzootic YFV genotype I lineages (designated as old-lineages) co-circulated and diversified while spread through different countries and Brazilian regions until the middle 1990s, following a pattern comparable to YFV genotype II lineages. However, we noted a remarkable change in this pattern from mid 1990s, when a reduction of genotype I genetic diversity occurred coinciding with the emergence of a new lineage (called modern-lineage) that rapidly disseminated throughout several countries replacing the old-ones. The modern-lineage probably emerged in Trinidad and Tobago at around 1977 and was the responsible for subsequent YFV outbreaks occurring in this country as well as in Brazil, Venezuela, Argentina and Colombia from mid 1990s onwards. Lineage replacement seems to be a common phenomenon in YFV genotype I evolution, as was observed previously during the replacement of the Old Pará lineages after the 1960s^12^.

Consistent with previous findings^13^, we found that enzootic maintenance (*in situ* evolution) seems to be the main mechanism shaping the evolutionary dynamics of YFV genotype I modern-lineage circulating in Trinidad and Tobago and Venezuela. The independent clustering of YFV sequences from the 2000–2001, 2008–2009 and 2016–2017 Brazilian outbreaks, by contrast, indicate that continuous sub-clade replacements and long-distance movements seems to be major driving forces of the evolution of the modern-lineage within this country. YFV lineage replacement was already reported between the 2000–2001 and 2008–2009 YF Brazilian outbreaks^10^. Here, we observed that the newly reported YFV sequences from the 2017 Southeastern Brazilian outbreak also probably resulted from the reintroduction of a modern-lineage YFV variant from Venezuela (or from some Brazilian endemic region), and not from the local persistence of modern-lineage variants previously circulating in the 2000–2001 or 2008–2009 Brazilian Southeastern outbreaks.

Previous phylogeographic studies suggested that YFV genotype I arose in Brazil at around the second half of 19th century^10^ and that Brazilian Northern region was the major viral source for surrounding regions and countries^4, 13, 15^. Here, we confirmed the central role played by the Northern Brazilian region in the spread of the YFV genotype I old-lineages between the 1960s and 1990s. From mid 1990s onwards, however, several different Brazilian regions and countries seem to have contributed to the spread of the YFV genotype I modern-lineage. Trinidad and Tobago was pointed as the primary source of YFV modern-lineage dissemination to Venezuela and Northern-Brazil, while secondary disseminations of this YFV lineage were detected from Venezuela to Northern and Southeastern Brazil, from Northern to Central-Western Brazil, from Central-Western to Northeastern, Southeastern and Southern Brazil and from Southeastern Brazil to Southern Brazil and Northern Argentina.

One important limitation of this study is the lack of geographically and temporarily balanced YFV datasets. In this sense, the dataset herein used presents a sharp drop in the number and proportion of Northern Brazilian sequences towards the present. Thus, the conclusion that the Northern Brazilian region was not the major source of YFV genotype I in America from middle 1990s onwards need to be taken with caution. Similarly, our analysis pointed to a direct viral dissemination from Venezuela to the Southeastern Brazilian region as the source of the 2017 Brazilian outbreak. However, given the remarkable long branch length separating the 2017 Brazilian sequences from its closest Venezuelan sequences, we could not rule out the existence of intermediate viral migration steps involving the North and Central-West Brazilian regions that were not recovered because temporal and geographical gaps in our data. As more YFV Brazilian sequences from both endemic and non-endemic regions become available, especially from recent YFV epizootic episodes occurring during the 2000s^2^, the dissemination pattern of the YFV modern-lineage in South America will be inferred with greater precision.

Previous estimates of the YFV population dynamics in South America have suggested that genotype I experienced a population growth rate with an extremely low epidemic doubling time (>20 years), while genotype II exhibited a constant population size, both consistent with epidemics dominated by sylvatic transmission^3^. These estimates were based on small sequence datasets covering only the YFV genetic diversity existing in the Americas until the year 2000. According to our reconstructions, the YFV genotype I went through an initial exponential growth phase between 1935 and 1950, followed by a period of *N*_e_ stabilization between 1950 and 1985. A subsequent reduction in the *N*_e_ of this YFV genotype was observed between 1985 and 2010 that seems to be explained by the replacement of old-lineages by a modern-lineage with a much lower *N*_e_ and by the confounding effect of geographic population subdivision as consequence of the more frequent sampling of epidemiologically linked sequences from recent YFV outbreaks^20, 21^. The demographic history reconstructed for YFV genotype II evidence a short exponential growth phase between 1955 and 1965, after which the *N*_e_ remains roughly stable up to the most recent time, with only small fluctuations between 1980 and 1995 that coincide with the spread of this genotype in Bolivia^14^. These results support that both YFV American genotypes displayed complex demographic patterns with significant temporal fluctuations in the *N*_e_ over time that were probably driven by both viral disseminations into new areas and viral lineage replacements.

It has been suggested that YFV is genetically stable and evolves slowly in comparison to other arboviruses^22^. Our search for genetic signatures in the ancestral nodes of genotype I, however, detected six amino acid substitutions fixed over an estimated interval of 55 years between the middle 1930s and late 1980s. Some of these substitutions were located in protein domains associated with important viral functions. The substitutions A79V and K82R are placed in the fourth α-helix of C protein, which has been associated with RNA binding, dimer formation, protein stability and infectious particle production^23, 24^. The substitution F2137L is positioned at N-terminal amphipathic helix of the NS4A protein, and a Leucine residue in that position of NS4A of Dengue virus type 2 was shown that could contribute to the protein oligomerization^25^. Finally, the amino acid substitutions A2736T (located in the guanylyltransferase/methyltransferase domain) and R3341K (located in the RNA dependent RNA polymerase domain) might modulate the NS5 activity since it was demonstrated biochemically that these domains interact tightly^26^. We also detected a large number of amino acid mutations (*n* = 35) that arose during recent dispersion of modern-lineage (subclade 1E) in Brazil and Venezuela, which could reflect more frequent viral replication cycles in large susceptible populations of primates from non-endemic regions and/or selective pressures for new viral variants with specific phenotypic characteristics. Of note, most (91%) amino acid substitutions detected in YFV modern-lineage accumulated within non-structural proteins that have been pointed as relevant selection targets in evolution of Flaviviruses^27, 28^.

In summary, we describe a dramatic change in the genetic diversity of the YFV genotype I in the Americas during the 1990s, associated with the dissemination of a new viral lineage (modern-lineage) that replaced the old ones in endemic areas and also spread to non-endemic South American regions. Trinidad and Tobago seems to be the most probably source of the YFV genotype I modern-lineage from where it spread to South American countries, originating several YFV outbreaks during the 21^st^ century. The recent 2017 Brazilian outbreak seems to be the result of a new reintroduction of the YFV modern-lineage into the Southeastern region, but more sequences from this outbreak are needed to confirm this hypothesis. It is not clear whether the successful dissemination of YFV genotype I modern lineage was driven by stochastic and/or adaptive factors. Several amino acid substitutions within non-structural proteins, however, have been fixed during dissemination of YFV modern-lineage and their potential impact on viral fitness, transmissibility and virulence deserves further investigation.

## Material and Methods

### YFV datasets

We collected all complete YFV genome sequences and prM/E gene sequences (654 nt in length) of American origin with known date of isolation that were available in GenBank (www.ncbi.nlm.nih.gov). Noncoding regions were removed from complete genomes, retaining only the complete polyprotein open reading frame (10,239 nt in length) for subsequent analyses. American YFV complete genome and prM/E sequences were manually aligned with reference YFV sequences from Africa and with vaccine strains obtained from GenBank and subsequent subjected to Maximum Likelihood (ML) phylogenetic analysis. ML phylogenetic trees were inferred with the PhyML program^29^, using the best-fit model of nucleotide substitution selected using the jModelTest program^30^. Heuristic tree search was performed employing the SPR branch-swapping algorithm and the reliability of the phylogenies was estimated with the approximate likelihood-ratio test (aLRT)^31^. Sequences that grouped with vaccine strains or within African genotypes (Supplementary Figures S1 and S3) and divergent sequences that appeared to have accumulated excessive mutations, as revealed by the linear regression analysis of the root-to-tip distances against sampling time method implemented in Tempest^32^, were removed. This resulted in a final data set of 22 YFV complete genomes and 137 prM/E sequences from nine American countries spanning a 63-year period (Supplementary Table S4). All selected YFV sequences were confirmed as non-recombinant using the Recombination Detection Program (RDP) v4.9^33^. Recombination analysis was performed using the methods RDP^34^, GENECONV^35^, MaxChi^36^, Chimaera^37^, BootScan^38^, SiScan^39^ and 3Seq.^40^ with their default settings. Only statistically significant (*P* < 0.05) events supported by at least two methods were considered.

### Phylodynamics analyses

The rate of nucleotide substitution, the time to the most recent common ancestor (T_MRCA_), the spatial diffusion pattern and the demographic dynamics of the YFV genotypes I and II in the Americas were jointly estimated using the Markov chain Monte Carlo (MCMC) algorithms implemented in the BEAST v1.8.3 package^41, 42^ with BEAGLE^43^ to improve run-time. The evolutionary and demographic process were directly estimated for each YFV genotype from the sampling dates of the prM/E sequences using the best-fit nucleotide substitution model, a relaxed uncorrelated lognormal molecular clock model^44^, and a Bayesian Skyline coalescent tree prior^45^. Migration events throughout the phylogeny were reconstructed using a reversible discrete phylogeographic model^46^ with a CTMC rate reference prior^47^. A discrete state was assigned for each sequence, corresponding to the country or country-region (Brazilian sequences) of isolation. Comparison between coalescent demographic models was performed using the log marginal likelihood estimation (MLE) based on path sampling (PS) and stepping-stone sampling (SS) methods^48^. MCMC were run sufficiently long to ensure stationarity and convergence. Uncertainty of parameter estimates were assessed after excluding the initial 10% of the run by calculating the Effective Sample Size (ESS) and the 95% Highest Probability Density (HPD) values, respectively, using TRACER v1.6^49^ program. The programs TreeAnnotator v1.7.5^41^ and FigTree v1.4.0^50^ were used to summarize the posterior tree distribution and to visualize the annotated Maximum Clade Credibility (MCC) tree, respectively.

### Reconstruction of ancestral YFV genotype I sequences

To map amino acid changes that were fixed during evolution of YFV genotype I, complete CDS were reconstructed at key internal nodes of the American YFV complete genome phylogeny using BEAST v1.8.3 package^41^. The temporal scale was directly inferred from the sampling date of the sequences applying the GTR+ I+ Γ^4^ substitution model selected using the jModelTest program, a relaxed uncorrelated lognormal molecular clock model and a Bayesian Skyline coalescent tree prior. After run convergence (10 million MCMC steps) the consensus complete CDS sequence for each key ancestral node were computed using the R package SeqinR^51^. The MCC tree was reconstructed as previously described.

## Electronic supplementary material

Supplementary Information
