# Supplementary material for: Phylodynamics of Yellow Fever Virus in the Americas: new insights into the origin of the 2017 Brazilian outbreak
Source: Sci Rep. 2017 Aug 7;7:7385. doi: 10.1038/s41598-017-07873-7 (PMC5547128; doi:10.1038/s41598-017-07873-7)
Supplement: Supplementary file 1 — Supplementary Information [file 41598_2017_7873_MOESM1_ESM.pdf]

Supplementary information

Phylodynamics of Yellow Fever Virus in the Americas: new insights into the origin of the 2017 Brazilian outbreak

Daiana Mir[1], Edson Delatorre[*1], Myrna Bonaldo[2], Ricardo Lourenço-de-Oliveira[3], Ana Carolina Vicente[4] and Gonzalo Bello[1]

1. Laboratório de AIDS e Imunologia Molecular, Instituto Oswaldo Cruz, Fiocruz, Rio de Janeiro, Brazil.
2. Laboratório de Biologia Molecular de Flavivírus, Instituto Oswaldo Cruz, Fiocruz, Rio de Janeiro, Brazil.
3. Laboratório de Mosquitos Transmissores de Hematozoários, Instituto Oswaldo Cruz, Fiocruz, Rio de Janeiro, Brazil.
4. Laboratório de Genética Molecular de Microrganismos, Instituto Oswaldo Cruz, Fiocruz, Rio de Janeiro, Brazil.
* Corresponding author: edsonod@ioc.fiocruz.br/delatorre.ioc@gmail.com

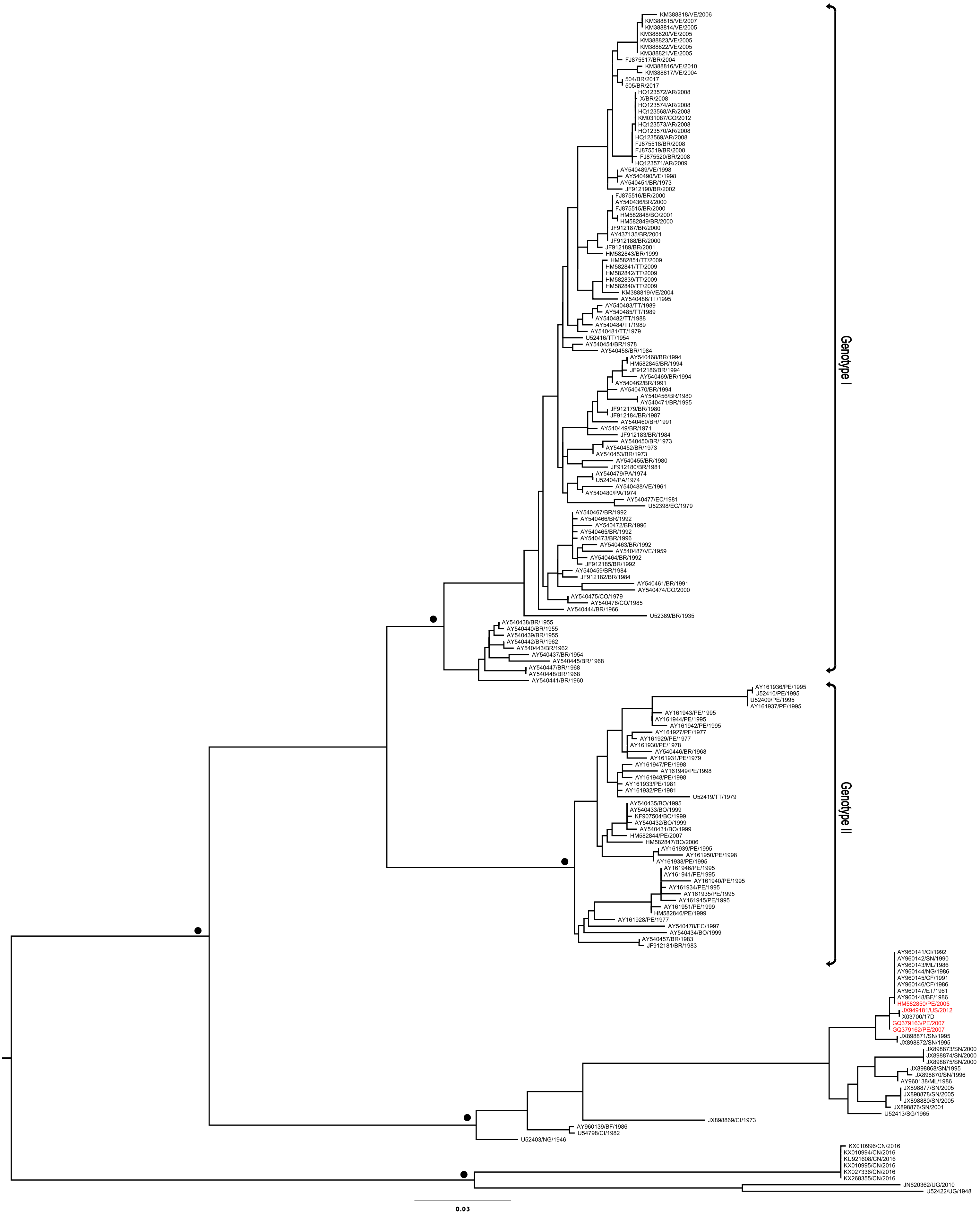

Supplementary Figure S1. Maximum likelihood phylogeny of YFV prM/E gene sequences. The aLRT support value of each indicated clade (dark full circle) was >0.8. American sequences that grouped with vaccine strain 17D are highlighted in red. The branch lengths are drawn to scale with bar at the bottom indicating nucleotide substitutions per site.

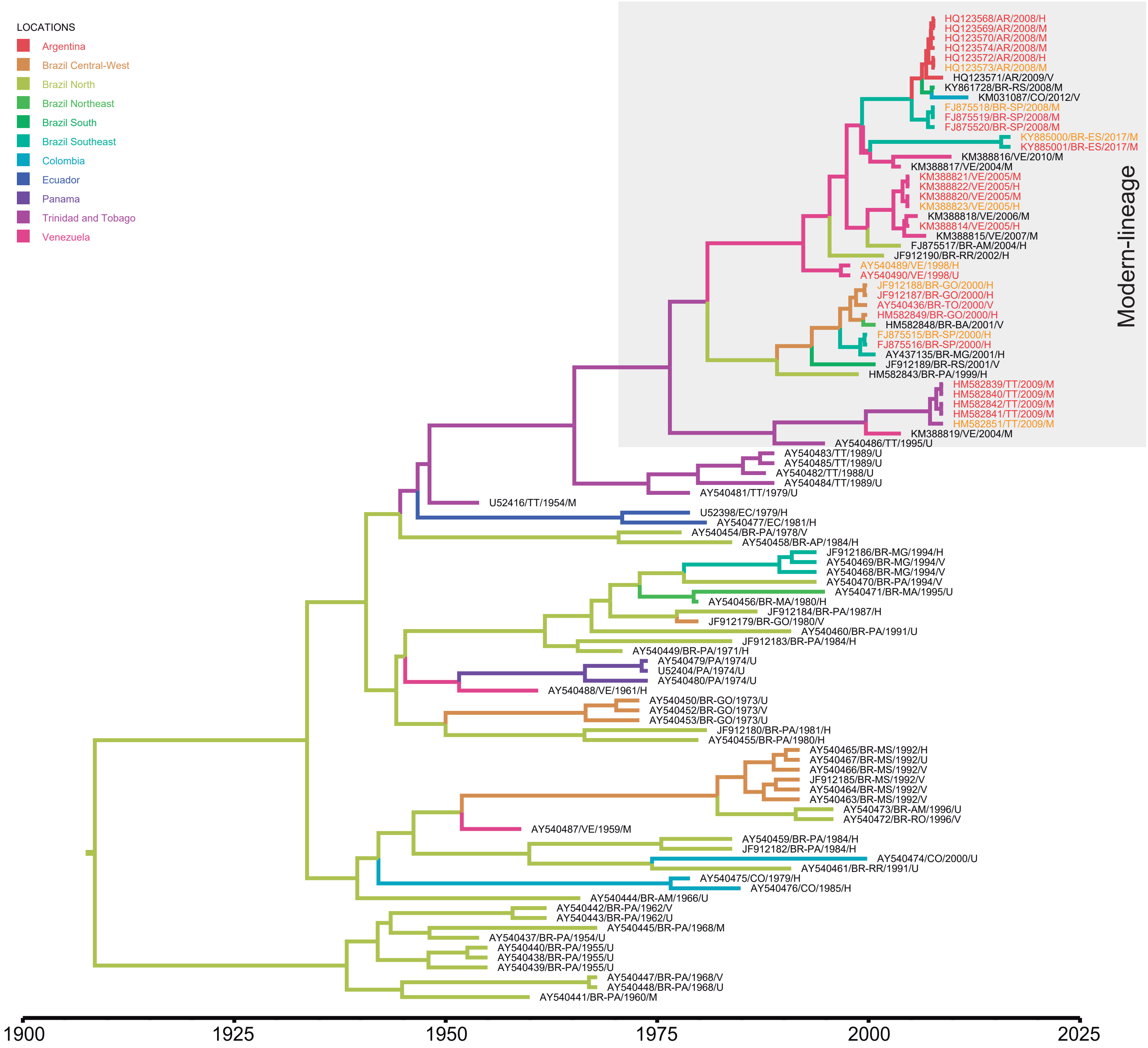

Supplementary Figure S2. Time-scaled Bayesian MCC phylogeographic tree of the YFV genotype I prM/E gene sequences. Branches are colored according to the most probable location state of their descendent nodes as indicated at the legend (top left). Tip labels of the modern-lineage (gray shaded area) sequences are colored indicating those epidemiologically related and sampled at the same YFV outbreak (red) and the one that was chosen as the representative of the location and year (orange). All horizontal branch lengths are drawn to a scale of years. The tree is automatically rooted under the assumption of a relaxed molecular clock.

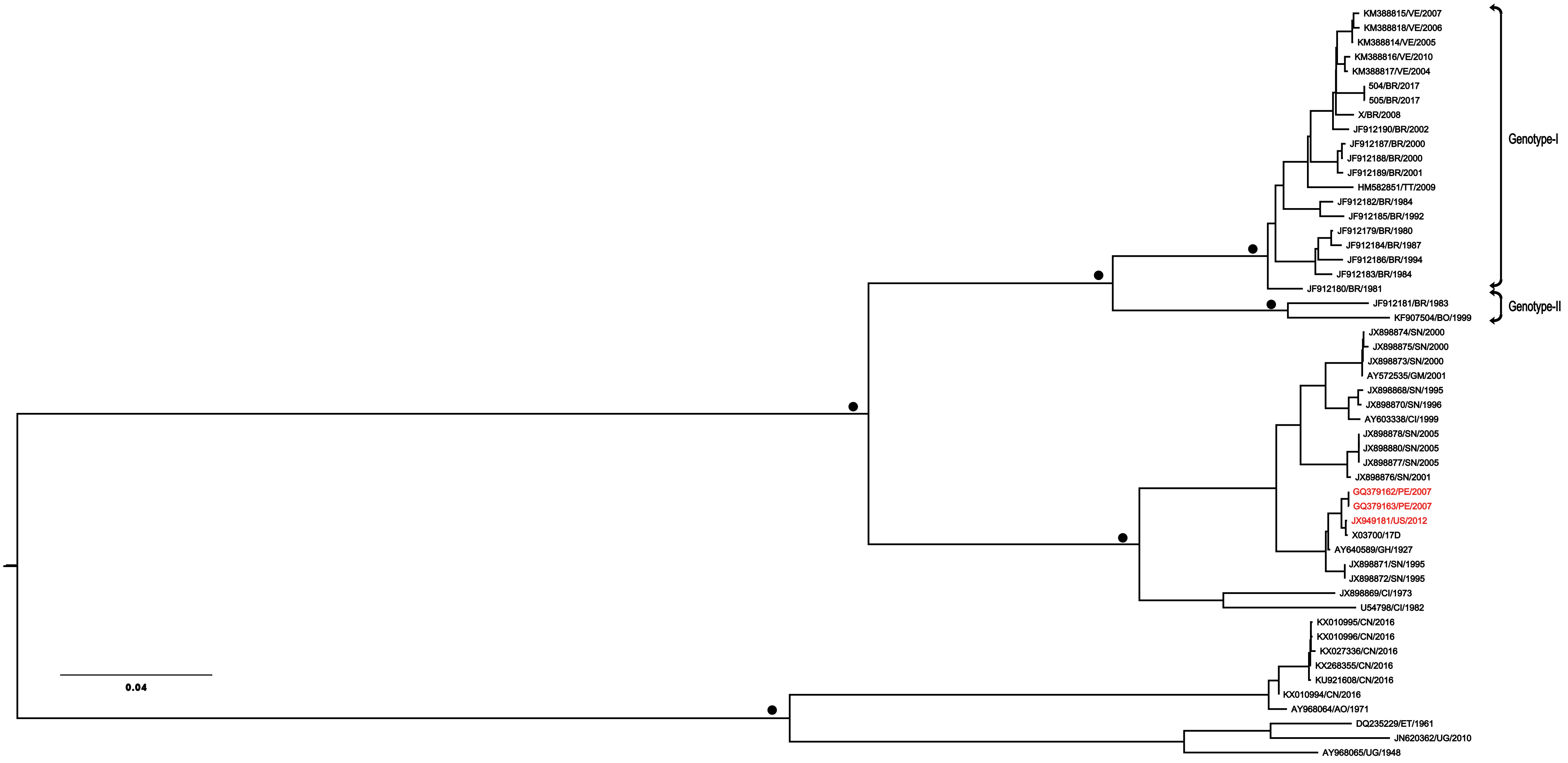

Supplementary Figure S3. Maximum likelihood phylogeny of YFV complete coding region sequences. The aLRT support value of each indicated clade (dark full circle) was = 1.0. American sequences that grouped with vaccine strain 17D are highlighted in red. The branch lengths are drawn to scale with bar at the bottom indicating nucleotide substitutions per site.

Supplementary Table S1. Best fit demographic model for YFV genotype II prM/E dataset.

Clade	Coalescent model	PS Log ML	Models compared	Log BF	SS Log ML	Models compared	Log BF
YFV-GII	Exponential	-2472.9	-	-	-2473.0	-	-
	BSKY	-2475.2	Exponential/BSKY	2.3	-2475.5	Exponential/BSKY	2.5
	Constant	-2480.6	Exponential/Constant	7.7	-2480.7	Exponential/Constant	7.7
			BSKY/Constant	5.4		BSKY/Constant	5.2
	Expansion	-2480.3	Exponential/Expansion	7.4	-2480.5	Exponential/Expansion	7.5
			BSKY/Expansion	5.1		BSKY/Expansion	5.0
	Logistic	-2481.1	Exponential/Logistic	8.2	-2481.5	Exponential/Logistic	8.5
			BSKY/Logistic	5.9		BSKY/Logistic	6.0

Log marginal likelihood (ML) estimates for the Bayesian skyline, logistic, exponential and expansion growth demographic models obtained using the path sampling (PS) and stepping-stone sampling (SS) methods. The Log Bayes factor (BF) is the difference of the Log ML between of alternative (H1) and null (H0) models (H1/H0). Log BFs > 3 indicates that model H1 is more strongly supported by the data than model H0.

Supplementary Table S2 - Polymorphisms found in the reconstructed ancestral YFV polyprotein open reading frame

Name	Type	CDS Position	CDS Codon Number	Polymorphism Type	Mature peptide	Change	Codon position	Codon Change	AA change	Nodes
P_1	Synonymous	162	54	transition	C	T -> C	3	AAT -> AAC		N2
P_2	Nonsynonymous	236	79	transition	C	C -> T	2	GCT -> GTT	A -> V	N3
P_3	Nonsynonymous	245	82	transition	C	A -> G	2	AAG -> AGG	K -> R	N3
P_4	Synonymous	318	106	transition	aC	G -> A	3	TTG -> TTA		N2
P_5	Synonymous	324	108	transition	aC	G -> A	3	GTG -> GTA		N3
P_6	Synonymous	331	111	transition	aC	C -> T	1	CTG -> TTG		N3
P_7	Synonymous	375	125	transition	prM	G -> A	3	GTG -> GTA		N2
P_8	Synonymous	432	144	transition	prM	T -> C	3	TTT -> TTC		N2
P_9	Synonymous	447	149	transition	prM	T -> C	3	GGT -> GGC		N3
P_10	Synonymous	513	171	transition	prM	T -> C	3	CCT -> CCC		N3
P_11	Synonymous	549	183	transition	prM	T -> C	3	GAT -> GAC		N2
P_12	Synonymous	558	186	transition	prM	T -> C	3	TGT -> TGC		N3
P_13	Synonymous	693	231	transition	M	C -> T	3	ACC -> ACT		N2
P_14	Synonymous	741	247	transition	M	A -> G	3	GTA -> GTG		N2
P_15	Synonymous	753	251	transition	M	T -> C	3	TTT -> TTC		N3
P_16	Synonymous	963	321	transition	E	T -> C	3	CCT -> CCC		N2
P_17	Synonymous	978	326	transition	E	G -> A	3	CTG -> CTA		N2
P_18	Synonymous	1.020	340	transition	E	A -> G	3	GAA -> GAG		N3
P_19	Synonymous	1.056	352	transition	E	T -> C	3	AAT -> AAC		N3
P_20	Synonymous	1.131	377	transition	E	T -> C	3	TGT -> TGC		N2
P_21	Synonymous	1.146	382	transition	E	T -> C	3	TCT -> TCC		N3
P_22	Synonymous	1.174	392	transition	E	C -> T	1	CTG -> TTG		N3
P_23	Synonymous	1.221	407	transition	E	T -> C	3	GCT -> GCC		N3
P_24	Nonsynonymous	1.385	462	transition	E	A -> G	2	AAG -> AGG	K -> R	N2
P_25	Synonymous	1.428	476	transition	E	C -> T	3	AGC -> AGT		N3
P_26	Synonymous	1.647	549	transition	E	T -> C	3	GTT -> GTC		N2
P_27	Synonymous	1.677	559	transition	E	C -> T	3	TAC -> TAT		N2
P_28	Synonymous	1.782	594	transition	E	T -> C	3	AAT -> AAC		N3
P_29	Synonymous	1.833	611	transition	E	G -> A	3	AAG -> AAA		N3
P_30	Synonymous	1.851	617	transition	E	C -> T	3	ATC -> ATT		N3
P_31	Synonymous	1.857	619	transition	E	G -> A	3	GTG -> GTA		N3
P_32	Synonymous	1.899	633	transition	E	T -> C	3	ATT -> ATC		N2
P_33	Synonymous	1.929	643	transition	E	T -> C	3	ACT -> ACC		N2
P_34	Synonymous	1.959	653	transition	E	C -> T	3	AAC -> AAT		N2
P_35	Synonymous	1.974	658	transition	E	C -> T	3	GAC -> GAT		N3
P_36	Synonymous	1.977	659	transition	E	C -> T	3	AGC -> AGT		N2
P_37	Synonymous	1.992	664	transition	E	A -> G	3	GGA -> GGG		N3
P_38	Synonymous	2.008	670	transition	E	T -> C	1	TTG -> CTG		N2
P_39	Synonymous	2.055	685	transition	E	C -> T	3	TTC -> TTT		N3
P_40	Synonymous	2.058	686	transition	E	C -> T	3	ACC -> ACT		N2
P_41	Synonymous	2.118	706	transition	E	C -> T	3	GGC -> GGT		N3
P_42	Synonymous	2.154	718	transversion	E	A -> C	3	GGA -> GGC		N2
P_43	Synonymous	2.223	741	transition	E	T -> C	3	ATT -> ATC		N2
P_44	Synonymous	2.436	812	transition	NS1	T -> C	3	TAT -> TAC		N3
P_45	Synonymous	2.503	835	transition	NS1	T -> C	1	TTG -> CTG		N2
P_46	Synonymous	2.532	844	transition	NS1	A -> G	3	GAA -> GAG		N3
P_47	Synonymous	2.568	856	transition	NS1	C -> T	3	ATC -> ATT		N2
P_48	Synonymous	2.577	859	transition	NS1	G -> A	3	GAG -> GAA		N3
P_49	Synonymous	2.625	875	transition	NS1	C -> T	3	TAC -> TAT		N2
P_50	Synonymous	2.643	881	transition	NS1	A -> G	3	CCA -> CCG		N2
P_51	Synonymous	2.673	891	transition	NS1	T -> C	3	TAT -> TAC		N3
P_52	Synonymous	2.829	943	transition	NS1	T -> C	3	ACT -> ACC		N3
P_53	Synonymous	2.880	960	transition	NS1	T -> C	3	TCT -> TCC		N3
P_54	Synonymous	2.976	992	transition	NS1	C -> T	3	CAC -> CAT		N2
P_55	Synonymous	2.982	994	transition	NS1	G -> A	3	CTG -> CTA		N2
P_56	Synonymous	3.219	1.073	transition	NS1	A -> G	3	GGA -> GGG		N3
P_57	Synonymous	3.339	1.113	transition	NS1	C -> T	3	ATC -> ATT		N2
P_58	Synonymous	3.483	1.161	transition	NS2a	A -> G	3	CTA -> CTG		N2
P_59	Synonymous	3.486	1.162	transition	NS2a	C -> T	3	GTC -> GTT		N2
P_60	Synonymous	3.568	1.190	transition	NS2a	T -> C	1	TTG -> CTG		N3
P_61	Synonymous	3.616	1.206	transition	NS2a	T -> C	1	TTG -> CTG		N2
P_62	Synonymous	3.687	1.229	transition	NS2a	G -> A	3	CGG -> CGA		N3
P_63	Synonymous	3.696	1.232	transition	NS2a	T -> C	3	CTT -> CTC		N2
P_64	Synonymous	3.735	1.245	transition	NS2a	A -> G	3	TTA -> TTG		N3
P_65	Synonymous	3.771	1.257	transition	NS2a	T -> C	3	AAT -> AAC		N2
P_66	Synonymous	3.999	1.333	transition	NS2a	A -> G	3	CTA -> CTG		N2
P_67	Synonymous	4.008	1.336	transition	NS2a	A -> G	3	ACA -> ACG		N2
P_68	Synonymous	4.017	1.339	transition	NS2a	T -> C	3	TTT -> TTC		N3
P_69	Synonymous	4.206	1.402	transition	NS2b	G -> A	3	GTG -> GTA		N2
P_70	Synonymous	4.366	1.456	transition	NS2b	T -> C	1	TTG -> CTG		N3
P_71	Synonymous	4.392	1.464	transition	NS2b	C -> T	3	CAC -> CAT		N3
P_72	Synonymous	4.458	1.486	transition	NS3	A -> G	3	GGA -> GGG		N3
P_73	Synonymous	4.473	1.491	transition	NS3	C -> T	3	GAC -> GAT		N3
P_74	Synonymous	4.554	1.518	transition	NS3	A -> G	3	GGA -> GGG		N3
P_75	Synonymous	4.618	1.540	transversion	NS3	A -> C	1	AGA -> CGA		N2
P_76	Synonymous	4.635	1.545	transition	NS3	C -> T	3	GTC -> GTT		N3
P_77	Synonymous	4.680	1.560	transition	NS3	G -> A	3	GAG -> GAA		N2
P_78	Synonymous	4.767	1.589	transition	NS3	G -> A	3	GGG -> GGA		N2
P_79	Synonymous	4.788	1.596	transition	NS3	G -> A	3	CAG -> CAA		N3
P_80	Synonymous	4.809	1.603	transition	NS3	A -> G	3	AAA -> AAG		N2
P_81	Synonymous	4.857	1.619	transition	NS3	C -> T	3	AGC -> AGT		N3
P_82	Synonymous	4.977	1.659	transition	NS3	A -> G	3	GAA -> GAG		N3
P_83	Synonymous	5.082	1.694	transition	NS3	C -> T	3	CCC -> CCT		N2
P_84	Synonymous	5.179	1.727	transition	NS3	C -> T	1	CTA -> TTA		N2
P_85	Synonymous	5.205	1.735	transition	NS3	C -> T	3	GCC -> GCT		N2
P_86	Synonymous	5.244	1.748	transition	NS3	C -> T	3	GCC -> GCT		N2

P_87	Synonymous	5.256	1.752	transition	NS3	T -> C	3	GCT -> GCC		N3
P_88	Synonymous	5.275	1.759	transition	NS3	C -> T	1	CTG -> TTG		N3
P_89	Synonymous	5.328	1.776	transition	NS3	C -> T	3	CAC -> CAT		N2
P_90	Synonymous	5.442	1.814	transition	NS3	C -> T	3	TTC -> TTT		N2
P_91	Synonymous	5.520	1.840	transition	NS3	C -> T	3	ATC -> ATT		N2
P_92	Synonymous	5.568	1.856	transversion	NS3	T -> A	3	GCT -> GCA		N3
P_93	Synonymous	5.622	1.874	transition	NS3	G -> A	3	CTG -> CTA		N2
P_94	Synonymous	5.781	1.927	transition	NS3	G -> A	3	AGG -> AGA		N2
P_95	Synonymous	5.931	1.977	transversion	NS3	G -> T	3	CTG -> CTT		N2
P_96	Synonymous	5.967	1.989	transition	NS3	G -> A	3	AGG -> AGA		N2
P_97	Synonymous	6.123	2.041	transition	NS3	C -> T	3	GCC -> GCT		N2
P_98	Synonymous	6.247	2.083	transversion	NS3	A -> C	1	AGG -> CGG		N2
P_99	Synonymous	6.396	2.132	transition	NS4a	C -> T	3	GAC -> GAT		N2
P_100	Nonsynonymous	6.409	2.137	transition	NS4a	T -> C	1	TTC -> CTC	F -> L	N2
P_101	Synonymous	6.426	2.142	transition	NS4a	G -> A	3	GAG -> GAA		N2
P_102	Synonymous	6.453	2.151	transition	NS4a	A -> G	3	TTA -> TTG		N3
P_103	Synonymous	6.501	2.167	transition	NS4a	T -> C	3	GCT -> GCC		N2
P_104	Synonymous	6.564	2.188	transition	NS4a	C -> T	3	TCC -> TCT		N3
P_105	Synonymous	6.636	2.212	transition	NS4a	C -> T	3	TCC -> TCT		N2
P_106	Synonymous	6.654	2.218	transition	NS4a	T -> C	3	TTT -> TTC		N2
P_107	Synonymous	6.813	2.271	transition	NS4b	G -> A	3	GGG -> GGA		N3
P_108	Synonymous	6.861	2.287	transition	NS4b	C -> T	3	GAC -> GAT		N3
P_109	Synonymous	6.957	2.319	transition	NS4b	C -> T	3	GGC -> GGT		N2
P_110	Synonymous	7.026	2.342	transition	NS4b	C -> T	3	TTC -> TTT		N2
P_111	Synonymous	7.143	2.381	transition	NS4b	T -> C	3	CCT -> CCC		N2
P_112	Synonymous	7.149	2.383	transversion	NS4b	T -> A	3	ATT -> ATA		N3
P_113	Synonymous	7.233	2.411	transition	NS4b	T -> C	3	GTT -> GTC		N3
P_114	Synonymous	7.383	2.461	transition	NS4b	G -> A	3	GGG -> GGA		N3
P_115	Synonymous	7.392	2.464	transversion	NS4b	A -> C	3	ATA -> ATC		N3
P_116	Synonymous	7.401	2.467	transition	NS4b	C -> T	3	AAC -> AAT		N2
P_117	Synonymous	7.539	2.513	transition	NS5	T -> C	3	ACT -> ACC		N2
P_118	Synonymous	7.540	2.514	transition	NS5	T -> C	1	TTG -> CTG		N3
P_119	Synonymous	7.650	2.550	transition	NS5	A -> G	3	CTA -> CTG		N3
P_120	Synonymous	7.662	2.554	transition	NS5	G -> A	3	AAG -> AAA		N2
P_121	Synonymous	7.680	2.560	transition	NS5	T -> C	3	GCT -> GCC		N2
P_122	Synonymous	7.731	2.577	transition	NS5	T -> C	3	GTT -> GTC		N2
P_123	Synonymous	7.735	2.579	transition	NS5	C -> T	1	CTG -> TTG		N3
P_124	Synonymous	7.869	2.623	transition	NS5	A -> G	3	CAA -> CAG		N3
P_125	Synonymous	7.893	2.631	transition	NS5	T -> C	3	ACT -> ACC		N3
P_126	Synonymous	7.902	2.634	transition	NS5	T -> C	3	GAT -> GAC		N3
P_127	Synonymous	7.941	2.647	transition	NS5	C -> T	3	GAC -> GAT		N2
P_128	Synonymous	7.971	2.657	transition	NS5	T -> C	3	TCT -> TCC		N3
P_129	Synonymous	7.998	2.666	transition	NS5	G -> A	3	AGG -> AGA		N2
P_130	Synonymous	8.085	2.695	transversion	NS5	A -> T	3	CCA -> CCT		N3
P_131	Synonymous	8.136	2.712	transversion	NS5	T -> A	3	GTT -> GTA		N2
P_132	Synonymous	8.148	2.716	transition	NS5	T -> C	3	CCT -> CCC		N2
P_133	Synonymous	8.163	2.721	transversion	NS5	T -> A	3	TCT -> TCA		N2
P_134	Synonymous	8.187	2.729	transition	NS5	A -> G	3	TCA -> TCG		N3
P_135	Synonymous	8.199	2.733	transition	NS5	C -> T	3	AGC -> AGT		N2
P_136	Nonsynonymous	8.206	2.736	transition	NS5	G -> A	1	GCG -> ACG	A -> T	N3
P_137	Synonymous	8.469	2.823	transition	NS5	C -> T	3	AGC -> AGT		N3
P_138	Synonymous	8.499	2.833	transition	NS5	A -> G	3	AAA -> AAG		N3
P_139	Synonymous	8.674	2.892	transition	NS5	T -> C	1	TTG -> CTG		N2
P_140	Synonymous	8.811	2.937	transition	NS5	A -> G	3	AAA -> AAG		N2
P_141	Synonymous	8.904	2.968	transition	NS5	A -> G	3	AAA -> AAG		N3
P_142	Synonymous	8.934	2.978	transition	NS5	T -> C	3	AGT -> AGC		N2
P_143	Synonymous	8.964	2.988	transition	NS5	C -> T	3	GCC -> GCT		N2
P_144	Synonymous	8.994	2.998	transition	NS5	T -> C	3	TTT -> TTC		N2
P_145	Synonymous	9.195	3.065	transition	NS5	T -> C	3	AGT -> AGC		N2
P_146	Synonymous	9.211	3.071	transition	NS5	C -> T	1	CTA -> TTA/TTG		N2
P_147	Synonymous	9.213	3.071	transition	NS5	A -> G	3	TTA -> TTG		N3
P_148	Synonymous	9.246	3.082	transition	NS5	T -> C	3	AAT -> AAC		N2
P_149	Synonymous	9.342	3.114	transition	NS5	C -> T	3	ACC -> ACT		N2
P_150	Synonymous	9.375	3.125	transition	NS5	C -> T	3	GTC -> GTT		N2
P_151	Synonymous	9.384	3.128	transition	NS5	C -> T	3	ATC -> ATT		N3
P_152	Synonymous	9.456	3.152	transition	NS5	A -> G	3	AAA -> AAG		N2
P_153	Synonymous	9.495	3.165	transversion	NS5	G -> C	3	CTG -> CTC		N2
P_154	Synonymous	9.576	3.192	transition	NS5	C -> T	3	AAC -> AAT		N2
P_155	Synonymous	9.597	3.199	transition	NS5	G -> A	3	AAG -> AAA		N3
P_156	Synonymous	9.648	3.216	transition	NS5	A -> G	3	GTA -> GTG		N2
P_157	Synonymous	9.651	3.217	transition	NS5	T -> C	3	CCT -> CCC		N2
P_158	Synonymous	9.744	3.248	transition	NS5	A -> G	3	GGA -> GGG		N2
P_159	Synonymous	9.765	3.255	transition	NS5	C -> T	3	GGC -> GGT		N2
P_160	Synonymous	9.930	3.310	transition	NS5	A -> G	3	AAA -> AAG		N3
P_161	Synonymous	9.954	3.318	transition	NS5	T -> C	3	GAT -> GAC		N2
P_162	Nonsynonymous	10.022	3.341	transition	NS5	G -> A	2	AGG -> AAG	R -> K	N2
P_163	Synonymous	10.176	3.392	transition	NS5	T -> C	3	TAT -> TAC		N3

Supplementary Table S3 - Amino acid mutations with respect to ancestor N3 found in the modern subclade 1E sequences

Name	Type	CDS Position	CDS Codon Number	Polymorphism Type	Mature peptide	Change	Codon position	Codon Change	Amino Acid Change	Variant Sequences (Accession number)
M_1	Nonsynonymous	322	108	transition	aC	G -> A	1	GTA -> ATA	V -> I	KY885000; KY885001
M_2	Nonsynonymous	356	119	transition	aC	T -> C	2	GTT -> GCT	V -> A	KM388817
M_3	Nonsynonymous	772	258	transition	M	G -> A	1	GCC -> ACC	A -> T	KM388817
M_4	Nonsynonymous	3185	1062	transition	NS1	G -> A	2	AGT -> AAT	S -> N	KY861728
M_5	Nonsynonymous	3191	1064	transition	NS1	T -> C	2	GTT -> GCT	V -> A	KM388817
M_6	Nonsynonymous	3328	1110	transition	NS1	C -> T	1	CCT -> TCT	P -> S	KY861728
M_7	Nonsynonymous	3397	1133	transition	NS2a	A -> G	1	ATC -> GTC	I -> V	JF912190
M_8	Nonsynonymous	3448	1150	transition	NS2a	G -> A	1	GTA -> ATA	V -> I	KM388814; KM388815; KM388818
M_9	Nonsynonymous	3532	1178	transition	NS2a	A -> G	1	ATC -> GTC	I -> V	JF912190
M_10	Nonsynonymous	3554	1185	transition	NS2a	C -> T	2	ACA -> ATA	T -> I	KM388815
M_11	Nonsynonymous	3590	1197	transition	NS2a	A -> G	2	AAT -> AGT	N -> S	KM388815
M_12	Nonsynonymous	3937	1313	transversion	NS2a	T -> A	1	TCA -> ACA	S -> T	KM388814; KM388815; KM388818
M_13	Nonsynonymous	4444	1482	transition	NS2b	G -> A	1	GCC -> ACC	A -> T	KM388814; KM388815; KM388818
M_14	Nonsynonymous	4716	1572	transversion	NS3	G -> T	3	GAG -> GAT	E -> D	KY885000; KY885001
M_15	Nonsynonymous	4773	1591	transversion	NS3	C -> A	3	AAC -> AAA	N -> K	KM388818
M_16	Nonsynonymous	4814	1605	transition	NS3	G -> A	2	AGG -> AAG	R -> K	KY885000; KY885001
M_17	Nonsynonymous	4997	1666	transition	NS3	A -> G	2	CAG -> CGG	Q -> R	KY861728
M_18	Nonsynonymous	5035	1679	transition	NS3	A -> G	1	ATT -> GTT	I -> V	KM388815
M_19	Nonsynonymous	5432	1811	transition	NS3	G -> A	2	AGT -> AAT	S -> N	JF912190
M_20	Nonsynonymous	5504	1835	transversion	NS3	C -> A	2	ACA -> AAA	T -> K	KM388818
M_21	Nonsynonymous	6932	2311	transversion	NS4b	A -> T	2	CAC -> CTC	H -> L	KM388818
M_22	Nonsynonymous	7223	2408	transition	NS4b	A -> G	2	AAT -> AGT	N -> S	KM388815; KM388818
M_23	Nonsynonymous	7506	2502	transversion	NS4b	G -> T	3	AAG -> AAT	K -> N	KM388818
M_24	Nonsynonymous	7508	2503	transition	NS4b	C -> T	2	ACT -> ATT	T -> I	KM388814
M_25	Nonsynonymous	7802	2601	transition	NS5	G -> A	2	AGA -> AAA	R -> K	KY861728
M_26	Nonsynonymous	7820	2607	transition	NS5	A -> G	2	AAG -> AGG	K -> R	KY885000; KY885001
M_27	Nonsynonymous	7840	2614	transition	NS5	G -> A	1	GAA -> AAA	E -> K	KM388817
M_28	Nonsynonymous	7930	2644	substitution	NS5	GTG -> ATA	1	GTG -> ATA	V -> I	KY885000; KY885001
M_29	Nonsynonymous	8035	2679	transition	NS5	G -> A	1	GGC -> AGC	G -> S	KY885000; KY885001
M_30	Nonsynonymous	8408	2803	transition	NS5	A -> G	2	AAT -> AGT	N -> S	KY885000; KY885001; KM388818
M_31	Nonsynonymous	9091	3031	transition	NS5	G -> A	1	GCA -> ACA	A -> T	KM388816
M_32	Nonsynonymous	9446	3149	transition	NS5	T -> C	2	GTT -> GCT	V -> A	KY885000; KY885001
M_33	Nonsynonymous	9472	3158	transition	NS5	G -> A	1	GCT -> ACT	A -> T	KM388816
M_34	Nonsynonymous	9644	3215	transition	NS5	A -> G	2	AAT -> AGT	N -> S	KY885000; KY885001
M_35	Nonsynonymous	10033	3345	transition	NS5	G -> A	1	GAC -> AAC	D -> N	KY861728

Supplementary Table S4 - List of the GenBank accession numbers of the YFV sequences used in this study

Country	Region	Genbank accession number	Collection date
Argentina	-	HQ123568	2008
	-	HQ123569	2008
	-	HQ123570	2008
	-	HQ123572	2008
	-	HQ123573	2008
	-	HQ123574	2008
	-	HQ123571	2009
Bolivia	-	AY540435	1995
	-	AY540431	1999
	-	AY540432	1999
	-	AY540433	1999
	-	AY540434	1999
	-	KF907504	1999
	-	HM582847	2006
Brazil	BR-AM	AY540444	1966
		AY540473	1996
		FJ875517	2004
	BR-AP	AY540458	1984
	BR-BA	HM582848	2001
	BR-ES	KY885000	2017
		KY885001	2017
	BR-GO	AY540450	1973
		AY540452	1973
		AY540453	1973
		JF912179	1980
		HM582849	2000
		JF912187	2000
		JF912188	2000
	BR-MA	AY540456	1980
		AY540471	1995
	BR-MG	AY540468	1994
		AY540469	1994
		JF912186	1994
		AY437135	2001
	BR-MS	AY540463	1992
		AY540464	1992
		AY540465	1992
		AY540466	1992
		AY540467	1992
		JF912185	1992
	BR-PA	AY540437	1954
		AY540438	1955
		AY540439	1955
		AY540440	1955
		AY540441	1960

		AY540442	1962
		AY540443	1962
		AY540445	1968
		AY540446	1968
		AY540447	1968
		AY540448	1968
		AY540449	1971
		AY540454	1978
		AY540455	1980
		JF912180	1981
		AY540459	1984
		JF912182	1984
		JF912183	1984
		JF912184	1987
		AY540460	1991
		AY540470	1994
		HM582843	1999
	BR-RO	AY540457	1983
		JF912181	1983
		AY540472	1996
	BR-RR	AY540461	1991
		JF912190	2002
	BR-RS	JF912189	2001
		KY861728	2008
	BR-SP	FJ875515	2000
		FJ875516	2000
		FJ875518	2008
		FJ875519	2008
		FJ875520	2008
	BR-TO	AY540436	2000
Colombia	-	AY540475	1979
	-	AY540476	1985
	-	AY540474	2000
	-	KM031087	2012
Ecuador	-	U52398	1979
	-	AY540477	1981
	-	AY540478	1997
Panama	-	AY540479	1974
	-	AY540480	1974
	-	U52404	1974
Peru	-	AY161927	1977
	-	AY161928	1977
	-	AY161929	1977
	-	AY161930	1978
	-	AY161931	1979
	-	AY161932	1981
	-	AY161933	1981
	-	AY161934	1995
	-	AY161935	1995
	-	AY161938	1995

	-	AY161939	1995
	-	AY161940	1995
	-	AY161941	1995
	-	AY161942	1995
	-	AY161943	1995
	-	AY161944	1995
	-	AY161945	1995
	-	AY161946	1995
	-	AY161947	1998
	-	AY161948	1998
	-	AY161949	1998
	-	AY161950	1998
	-	AY161951	1999
	-	HM582846	1999
	-	HM582844	2007
Trinidad and Tobago	-	U52416	1954
	-	AY540481	1979
	-	U52419	1979
	-	AY540482	1988
	-	AY540483	1989
	-	AY540484	1989
	-	AY540485	1989
	-	AY540486	1995
	-	HM582839	2009
	-	HM582840	2009
	-	HM582841	2009
	-	HM582842	2009
	-	HM582851	2009
Venezuela	-	AY540487	1959
	-	AY540488	1961
	-	AY540489	1998
	-	AY540490	1998
	-	KM388817	2004
	-	KM388819	2004
	-	KM388814	2005
	-	KM388820	2005
	-	KM388821	2005
	-	KM388822	2005
	-	KM388823	2005
	-	KM388818	2006
	-	KM388815	2007
	-	KM388816	2010
